# The Service Capability of Primary Health Institutions under the Hierarchical Medical System

**DOI:** 10.3390/healthcare10020335

**Published:** 2022-02-10

**Authors:** Shuo Liu, Jintao Lin, Yiwen He, Junfang Xu

**Affiliations:** School of Public Health, Zhejiang University School of Medicine, Hangzhou 310058, China; a1147465914@163.com (S.L.); ljt181151104@163.com (J.L.); 3190104273@zju.edu.cn (Y.H.)

**Keywords:** primary health institution, service capability, hierarchical medical system

## Abstract

Background: Primary health institutions (PHIs) are the foundation of the whole health system and the basic link to achieve the goal of all people enjoying primary health care. However, the service capability of primary health institutions is not under the hierarchical medical system. Method: Data were collected from the *China Health Statistics Yearbook* between 2014 and 2020. PHIs included community health centres, community health stations, and township hospitals in our study. The service capability of primary health institutions was analysed from the perspective of structure, process, and results. Structure capability was evaluated using the number of beds, number of personnel, number of health technicians, and proportion of the number of personnel in PHIs accounting for the total number of health personnel. Process capability was evaluated using the number of general practitioners. The number of outpatients and inpatients, medical income, the proportion of drug income, and the average number of patients and beds served by physicians in PHIs per day were employed to evaluate the resulting capability. Results: From 2014 to 2020, the number of community health service centres/stations increased, while the number of township health centres decreased. In the aspect of structure capability, the total number of personnel and health technicians in community health centres/stations and township hospitals both increased during 2014 and 2020. However, the increasing rate in PHIs was a little bit less than that of general medical institutions. The proportion of male health technicians in community health centres and township hospitals both decreased, while the proportion of female technicians in both increased. From 2014 to 2020, the number of beds in PHIs also increased from 138.12 × 10^4^ to 164.94 × 10^4^. However, the proportion of beds in PHIs accounting for the total number of beds in medical institutions decreased. For the resulting capability, from 2014 to 2019, the proportion of diagnosis and treatment times in PHIs decreased from 57.41% to 51.96%, although it increased in 2020. The proportion of inpatients in PHIs decreased from 20.03% to 16.11%. From 2014 to 2020, the utilisation rate of hospital beds in PHIs decreased (from 55.6% to 34% for community health centres and 60.5% to 53.6% for township hospitals). The average daily bed days of doctors in township hospitals was higher than that of doctors in community health service centres. However, the average medical cost of outpatients and the per capita medical cost of inpatients in community health service centres were higher than in township hospitals. Conclusion: In recent years, although the service capability showed an increasing trend in PHIs, the growth rate was lower than the general health institutions. The utilisation rates of PHIs, including beds and physicians, were decreased. Among PHIs, the utilisation in township hospitals was higher than in community health centres with a relatively low price. Under the hierarchical medical system and normalisation period of the COVID-19 epidemic, it is important to improve the service capability to achieve its goal of increasing PHI utilisation and decreasing secondary and tertiary hospital utilisation.

## 1. Introduction

Chronic diseases have become the biggest disease burden in China in the past 30 years. The primary goal of China’s health system is to prevent and control chronic diseases, especially among the elderly. This reflects the urgency of speeding up the capacity-building of primary health institutions (PHIs) [1,2] as the foundations of the whole health system, which undertake the responsibility of providing basic health services, including public health, to people [1]. The ageing and growing burden of chronic diseases in China also highlights the need for a continuous and coordinated system based on primary health services [2]. To improve the health service level of the hierarchical health system, health resources should be effectively provided to the primary institutions, so as to promote the optimisation and adjustment of the structure, function, and layout of the health system as a whole.

The importance of primary healthcare has also been highlighted after the pandemic of COVID-19 [3]. For example, more than 4 million personnel from PHIs across the country participated in preliminary screening, diagnosis, and referral, which has greatly alleviated the pressure faced by general hospitals [4]. In addition, the importance of PHIs is not only to be reflected in the public health of disease prevention, but also in the diagnosis and treatment of some common diseases, especially during the pandemic. Currently, during the epidemic normalisation period, all the patients with a fever can be found in time through the PHIs. The fever patients can be transferred to the fever clinics at the higher-level hospitals to receive timely and effective treatment, which has played a positive role in the effective control of COVID-19. Although the application of tiered medical services in the prevention and control of sudden epidemic situations is quite effective, some problems have also emerged, such as the capability of PHIs in China.

Continuous monitoring and health management of COVID-19 patients after they are discharged from the hospital are carried out by PHIs. The COVID-19 epidemic is a huge test for the construction of medical alliances encouraged by China in recent years, which fully exposes the weak service level of PHIs. Since the outbreak of the epidemic, a considerable number of PHIs have been confronted with problems such as insufficient medical supplies, insufficient manpower and skills, and loopholes in public information systems. They are basically not equipped with screening and isolation capabilities, making it difficult to implement screening and classified isolation for community residents.

The Chinese government has recently issued a series of policies to improve primary health care. On 4 December 2020, the National Health Commission of China issued the notice on deepening the “Internet plus Health”, and encouraged the development of the information system for PHIs [5]. On 24 May 2021, the State Council issued the 2021 key tasks for deepening the reform of the health system. One of the tasks is to accelerate the improvement of the basic infrastructure of community health centres and township hospitals [4]. It can be seen that the hierarchical medical system attaches unprecedented importance to the construction of the service capability of PHIs. At the same time, the implementation of a two-way referral policy, which requires the first diagnosis at PHIs, provides another chance to improve primary health care. The service capability of PHIs is directly related to the construction of the hierarchical health system and universal health coverage. Moreover, it also influences the social satisfaction of residents as a result of it often being difficult and expensive to see a doctor in China [6].

However, the current status of PHIs’ service capability or the impacts of hierarchical medical system construction on PHIs’ capability are unclear; for example, whether the capability of PHIs is increasing and in line with the goals of the hierarchical medical system. Scholars have performed a lot of research on the improvement paths of the medical service capacity of PHIs in China. However, most studies used one place or several indicators; the analysis of the change of the medical service capacity of PHIs from a comprehensive and national perspective is limited. To find the problems existing in the service of PHIs in China and provide evidence for improving the capability of primary health care, we aim to analyse the capability of PHIs from 2014 to 2020 from structural, process, and result perspectives.

## 2. Methods

### 2.1. Data Source

The related data of PHIs were collected from the *China Health Statistics Yearbook* [7] from 2014 to 2020, respectively, which included number of PHIs, personnel including health technicians in PHIs, number of health technicians in PHIs, beds and equipment above 10,000 yuan in medical institutions and PHIs, general practitioners in China, number of diagnoses and treatment times in PHIs, utilisation rate of hospital beds in PHIs, income of PHIs averaged by number of doctors’ visits per day in PHIs, average daily bed days of doctors in PHIs, and medical expenses of patients.

### 2.2. Measurement of Service Capability

In the study, PHIs included community health centres, community health stations, and township hospitals. The service capability of PHIs was divided into structural service capability, process service capability, and result service capability. The indexes measuring structural service capability included the number of health technicians in China, the number of primary health technicians, the proportion of primary health technicians (the number of primary health technicians in PHIs to the number of health technicians in total medical institutions), the number of beds in PHIs, the proportion of beds in PHIs (the number of beds in PHIs to the number of beds in total medical institutions), and the utilisation rate of beds in PHIs. Number of general practitioners was used to evaluate the process service capability of PHIs. The indexes of result service capability contained the number of outpatients and inpatients in PHIs, medical income of PHIs, the proportion of drug income (the income of drugs in PHIs to the total drug income of total medical institutions), and the average number of patients and beds served by physicians in PHIs per day.

### 2.3. Statistical Analysis

Descriptive statistical analysis (i.e., frequency and percentage) was used to analyse the structure, process, and outcome service capability indicators of PHIs from 2014 to 2020. The trend and comparative analysis of the service capability of PHIs from 2014 to 2020 were also conducted. All data analysis was based on the statistical software SPSS 23.0 (IBM, Armonk, NY, USA). Variables with *p* < 0.05 were considered as statistically significant.

## 3. Results

### 3.1. Structure Service Capability in PHIs from 2014 to 2020

**Figure 1:**
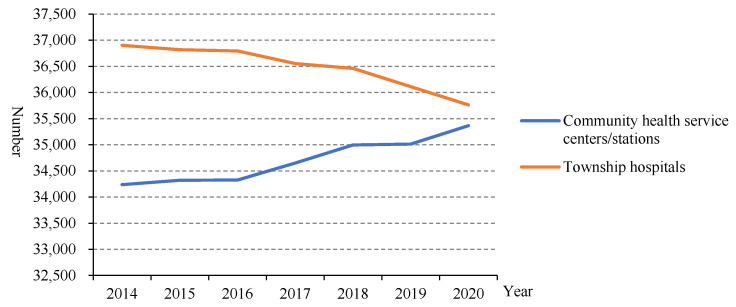
The number of PHIs in China from 2014 to 2020.

The number of community health service centres/stations increased from 34,238 in 2014 to 35,365 in 2020, while the number of township health centres decreased from 36,902 in 2014 to 35,762 in 2020 (Figure 1).

**Figure 2:**
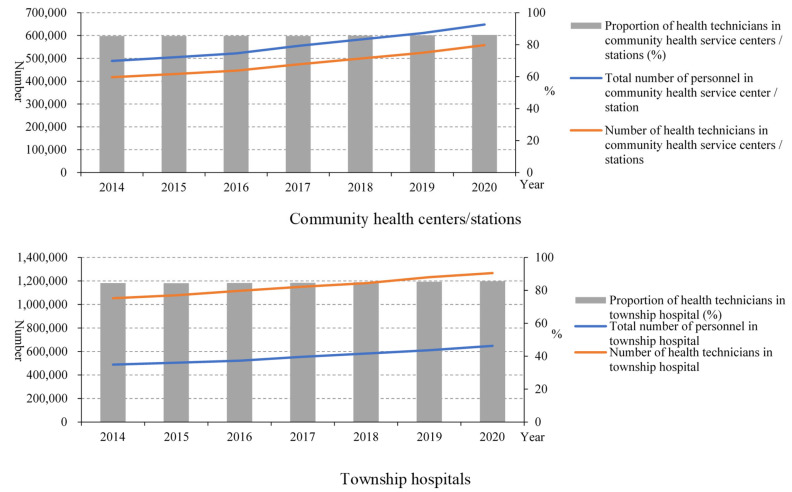
Personnel including health technicians in PHIs.

From 2014 to 2020, the total number of personnel and the number of health technicians in community health centres/stations both increased (Figure 2). The proportion of health technicians accounting for the total number of personnel in community health centres/stations remained almost unchanged before 2020 and increased slightly in 2020 (86.12%) compared to 2014 (85.42%). Similarly, the total number of personnel and the number of health technicians in township hospitals also increased, and the proportion of health technicians accounting for the total number of personnel in township hospitals remained almost unchanged before 2020, and increased slightly to 85.55% in 2020 compared to 84.45% in 2014.

**Figure 3:**
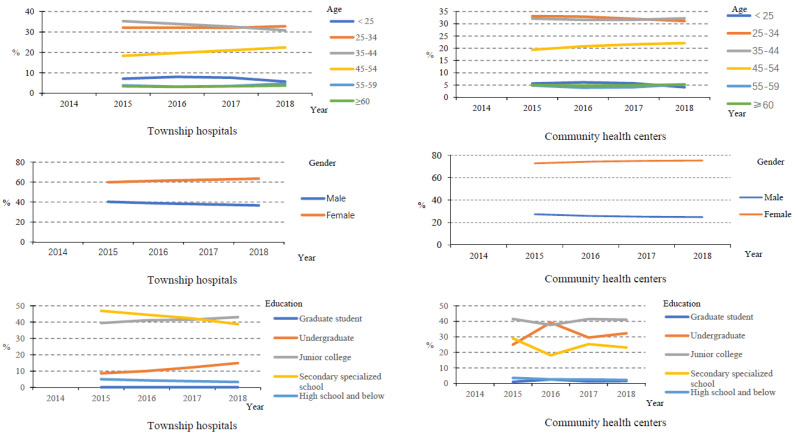
The characteristics of health technicians in PHIs.

From Figure 3, we can see that the number of health technicians younger than 45 years old was reduced, and the proportion of male health technicians in PHIs, including community health centres and township hospitals, also showed a decreasing trend, while the proportion of females increased. Moreover, most health technicians (~60%) had an education level lower than undergraduate.

**Figure 4:**
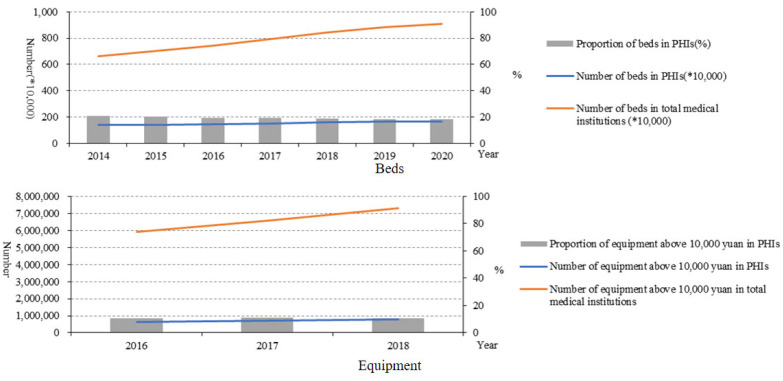
Beds and equipment above 10,000 yuan in medical institutions and PHIs.

Although the number of beds in PHIs also increased to 164.94 × 10^4^ in 2020 from 138.12 × 10^4^ in 2014, the proportion of beds in PHIs accounting for the total number of beds in medical institutions decreased from 20.92% in 2014 to 18.12% in 2020. The equipment valued above 10,000 RMB showed a similar trend with beds in the number and proportion (Figure 4).

### 3.2. Process Service Capability of PHIs

**Figure 5:**
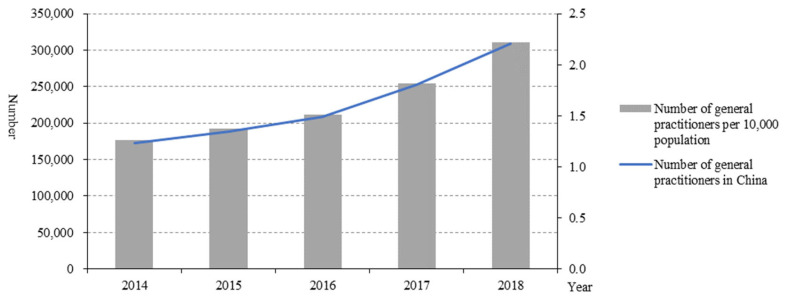
General practitioners in China from 2014 to 2018.

The number of general practitioners in China increased from 172,597 in 2014 to 308,740 in 2018 (Figure 5), as did the number of general practitioners per 10,000 people (1.26 in 2014 to 2.22 in 2018).

### 3.3. Result Service Capability of PHIs

**Figure 6:**
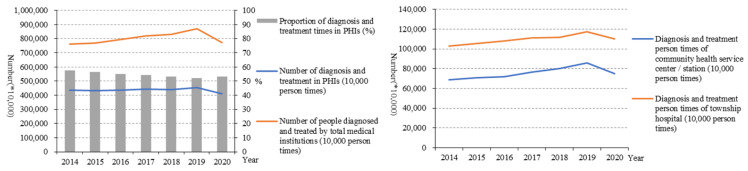
Number of diagnoses and treatments in PHIs (10,000 person-times).

From 2014 to 2019 (Figure 6), the number of diagnoses and treatment times by total medical institutions increased from 760,186.6 (10,000 person-times) to 872,000 (10,000 person-times), but it decreased to 774,000 (10,000 person-times) in 2020. For PHIs, the number of diagnoses and treatment declined from 453,087.1 (10,000 person-times) to 412,000 (10,000 person-times). Among PHIs, the number of diagnoses and treatment times in township hospitals was much higher than that of community health centres.

**Figure 7:**
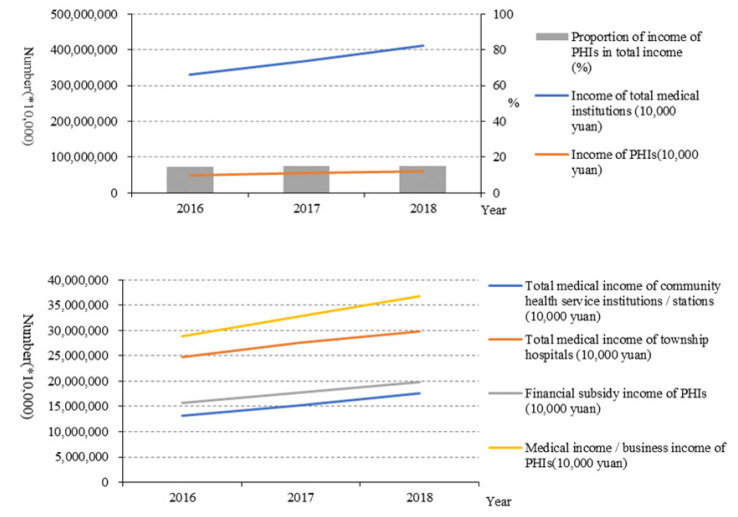
Income of PHIs (10,000 yuan).

From 2016 to 2018, the income of PHIs increased from 48,293,753 (10,000 yuan) to 61,246,366 (10,000 yuan), and the proportion of the income of PHIs accounting for the total income in medical institutions increased slightly from 14.56% to 14.9%. Among PHIs, the income in township hospitals was higher than that of community health centres. Moreover, the medical income in PHIs was much higher than government subsidies (Figure 7).

**Figure 8:**
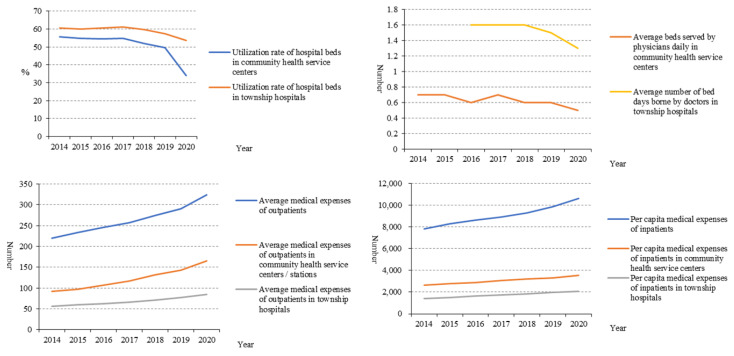
Beds utilisation, days served by doctors, and medical expenses of patients in PHIs.

Figure 8 shows that the utilisation of beds in township hospitals was also higher than that of community health centres. However, both showed a decreasing trend during 2014 and 2020. The bed days per doctor’s service also had a similar trend. The medical expenses per patient in PHIs were much lower than those of general medical institutions, and compared with community health centres, they were lower in township hospitals. However, the medical expenses per patient in PHIs increased in the last six years.

## 4. Discussion

Since the implementation of the new medical reform, the service capability of PHIs has been improved, especially by the structure service capability. In the number of PHIs, community health service centres increased, while the number of township hospitals decreased. This is related to the fast urbanisation, which means many rural areas turned to urban areas and rural populations migrated to cities in China in recent years [8]. From the point of view of the structural service capability of the PHIs, the structural service capability of the PHIs in China has been improved as a whole. From 2015 to 2020, the number of health technicians in PHIs gradually increased, but the proportion of health technicians remained almost unchanged. This is because the number of total staff in PHIs also increased. In addition, from 2015 to 2018, the health technicians in community health service centres mainly had junior college degrees and undergraduate degrees, and the health technicians in township health centres mainly had secondary school degrees and junior college degrees, while there were very few with graduate degrees, suggesting that the construction of the talent teams in PHIs urgently needs to be improved [9]. From 2014 to 2020, the number of beds in PHIs also increased year by year, but the proportion of beds in PHIs decreased year by year. Moreover, the proportion of equipment above 10,000 RMB in PHIs remained almost unchanged. The number of health technicians younger than 35 years old was reduced, which may be due to the number of young people choosing to be health technicians reducing gradually. The proportion of male health technicians in PHIs also showed a decreasing trend, while the proportion of females increased. This may be related to safe working practices and low salaries in primary health facilities. Such characteristics make women who need more care for their families choose this job. These results indicated that the government should pay more attention to the resource allocation of PHIs. Moreover, reviewing the course of the COVID-19 epidemic, we can also find that the improvement of PHIs is the “most economical and effective” measure to control infectious diseases. The capability of PHIs is also the key to realising tiered diagnosis and treatment. To sum up, the structural service capability of PHIs in China has been improved as a whole, but there are still deficiencies that need to be improved.

As the gatekeeper of primary medical care, the number of general practitioners in China increased. The first consultation system of general practitioners is the key to realising the function of the gatekeeper [10]. The establishment of the first consultation system of general practitioners can give full play to the core role of rational allocation and effective utilisation of health resources [11]. General practitioners guide patients to seek medical treatment in a scientific and orderly manner and on-demand [12]. This also showed that under the hierarchical health system, primary healthcare and its capabilities were gradually improved. However, due to the lack of data, it is temporarily impossible to analyse the contract services of general practitioners in PHIs.

From the point of result service capability, the progress of the result service capability of the PHIs in China was not strong, and the utilisation of diagnoses and treatment at the PHIs level was low, although evidence has shown that 80% of diseases can receive effective treatment in the PHIs. From 2014 to 2019, the number of diagnoses and treatment times in PHIs almost remained the same and decreased in 2020. In addition, from 2019 to 2020, the number of diagnoses and treatment times by total medical institutions decreased, too. This has to do with the fact that COVID-19 has kept people indoors [13]. In addition, the proportion of diagnoses and treatment times in PHIs accounting for the total also decreased. The low utilisation of PHIs may also affect whether the COVID-19 epidemic can be effectively controlled. The main reason for the low utilisation may be the relatively low capacity in PHIs. In addition, the development of PHIs still lags behind the development of general hospitals, and the relatively weak situation of PHIs has not been substantially improved, which has been clearly shown in our results regarding service capability.

Among PHIs, the number of diagnoses and treatment times in township hospitals was much higher than that of community health centres. One explanation is that people in rural areas are more likely to choose township hospitals over large urban hospitals due to traffic constraints [6]. What is more, the average daily bed days of doctors in PHIs also did not increase. These indicated that patients tended to seek treatment in the general hospitals rather than PHIs. The government financial subsidy income and medical income both increased year by year, which showed that the new medical reform attaches enough importance to the financial support of PHIs and has achieved initial results [14]. Although the income of PHIs in China has improved, in order to achieve long-term development, PHIs need to make good use of this income, improve service capability, and create more value [15]. All in all, the outcome service capability of PHIs in China has been improved on the whole, but the effect is weak, and the reform efforts need to be further strengthened [16,17,18,19]. On the one hand, they should promote the effective integration of medical resources and improve the sharing system of medical resources in the region to strengthen the ability of PHIs. On the other hand, they should improve the helper mechanism of an integrated health care system by encouraging hospital professionals working in the PHIs regularly.

This study was subjected to some limitations. First, some aspects may benefit a lot from a qualitative study rather than quantitative research. Second, considering the significant difference in geography and economic levels, the service capability of PHIs are different within China, but this study lacks regional analysis. Third, due to the lack of some data, the process service capability cannot be analysed in depth. Moreover, future studies may benefit from a deep statistics analysis.

## 5. Conclusions

Under the hierarchical health system and normalisation period of the COVID-19 epidemic, to improve the capability of primary health services, many measures have been issued, including PHIs’ functional orientation, talent team construction and financial compensation mechanism, structural service capability, process service ability, and the result service ability. These were improved, but on the whole, failed to achieve the expected effect, and some indicators even showed a downward trend, showing the weakening of medical service capabilities and the shrinking of medical service scope. This may significantly affect the carrying out of high-quality and effective health systems, as well as the emergency management of infectious diseases.

## Data Availability

All of the main data have been included in the results. Additional materials with details may be obtained from the corresponding author.
